# Supplementary material for: Predictors of neonatal hypothermia within six hours of birth and exploring preventive practices among post-natal mothers in Kilimanjaro region: Explanatory sequential mixed method protocol
Source: PLoS One. 2024 Nov 8;19(11):e0313432. doi: 10.1371/journal.pone.0313432 (PMC11548749; doi:10.1371/journal.pone.0313432)
Supplement: S1 File — (DOCX) [file pone.0313432.s001.docx]

**In-depth interview guide**

**Topics**

QN1: In your opinion, should neonate be kept warm after birth? Probe: Why? Is it possible to detect hypothermia after delivery to your neonates? How is it done? Have you performed, and how did you do it?

QN2: How do you keep your neonates warm after delivery? At home?  Probe; skin-to-skin contact, breastfeeding,

QN3: Can you share the challenges of keeping your neonates warm, how you cope with them,

Probe: Any support from family, community, and healthcare providers?
