# Supplementary material for: Predictors of neonatal hypothermia within six hours of birth and exploring preventive practices among post-natal mothers in Kilimanjaro region: Explanatory sequential mixed method protocol
Source: PLoS One. 2024 Nov 8;19(11):e0313432. doi: 10.1371/journal.pone.0313432 (PMC11548749; doi:10.1371/journal.pone.0313432)
Supplement: Attachment — Submitted filename: Rebuttal letter(Response to reviewer).docx [file pone.0313432.s003.docx]

**Tittle: Predictors of neonatal hypothermia within six hours of birth and exploring preventive practices among post-natal mothers in Kilimanjaro region: explanatory sequential mixed method protocol**

**Ref: Submission ID:** **PONE-D-12746**

**REVIEWER #2: AUTHOR'S RESPONSES TO COMMENTS**

| **S/N** | **REVIEWER’S COMMENT** | AUTHOR’S RESPONSE | **PG. NUMBER** |
| --- | --- | --- | --- |
| **1** | The introduction is relevant and satisfactory information about the topic is presented in an organized way, yet, the researcher needs to add more Introduction is relevant and satisfactory information about the topic is presented in an organized way, yet, the researcher needs to add more previous studies mainly related to prediction and preventive measures taken by mothers worldwide, not only in the African region., not only in the African region. | Previous studies mainly related to prediction and preventive measures taken by mothers worldwide, have been added to the introduction | Pg 5 of the revised study protocol |
| **2** | On page no 3: I suggest using "implications of study” instead of discussion. | No, change has been made since due to PLOS one journal guideline, hence the word discussion remained in the abstract as appeared before | Pg. 3 of the revised study protocol |
| **3** | Also, the researcher uses the term (interview to collect the qualitative data). It is better to use the term (Focus group discussing: FGD) as a preferable technique for the collection of this type of data. | This study intends to explore the individualised experience about the phenomenon and not a shared experience, therefore in-depth interview was opted for and not FGDs | Pg. 18-19 of the revised study protocol |
| **4** | Page 6: Researchers must add objective(s) related to (protocol) that researchers seek to achieve and develop. | To develop strategies to increase adherence to thermal care guidelines to reduce neonatal hypothermia among post-natal mothers. | Pg. 6 of the revised study protocol |
| **5** | The methods and materials are generally appropriate.  However, more elaboration in the analysis of the qualitative data is needed, and researchers should provide justification: | More details about the analysis of qualitative study have been revised | Pg. 20-21 of the revised study protocol |
| **6** | Why only two districts were chosen and how many districts are there in the country under study, as well as why only four health facilities? | Two districts were selected out of seven districts of the Kilimanjaro region because all districts share similar characteristics in terms of social structure and culture. Additionally, four health facilities were selected because of the equal availability of healthcare and a similar number of facility delivery thus sample size can be achieved at a minimum of four facilities. | Pg.9 of the revised study protocol |
| **7** | Page no 8: more expansion why researchers chose both (full-term & preterm neonates) as samples, it is recognized that heat loss risk factors differ between premature and full-term neonates. | The researcher has selected both full-term and preterm neonates because of getting diversification of the magnitude of the problem and  the development of evidence-based interventions and recommendations tailored to the unique needs of each population | Pg. 10 of the revised study protocol |
| **8** | Data collection tools: Researchers need to add how to test the validity and reliability of data collection tools | The reliability and validity of the tools have been revised in the study protocol | Pg.14 of the revised study protocol |
| **9** | Researchers will take (15) post-natal mothers as samples, so they need to add what precautions will be taken in case of postnatal mothers’ withdrawal from the study. Some references need to be updated | The study will take 15 postnatal women as a baseline sample size since, in qualitative research, there is no fixed sample size; the sample size is determined by data saturation as indicated in the revised study protocol but for the case of withdrawal from the study, the replacement of the participants will be used as a mechanism to maintain the sample size in case of withdraw | Pg. 18-19 of the revised study protocol |
| **10** | Some references need to be updated | All References revised and all are updated | Pg 26-29 of the revised study protocol |
