# Supplementary material for: Predictors of neonatal hypothermia within six hours of birth and exploring preventive practices among post-natal mothers in Kilimanjaro region: Explanatory sequential mixed method protocol
Source: PLoS One. 2024 Nov 8;19(11):e0313432. doi: 10.1371/journal.pone.0313432 (PMC11548749; doi:10.1371/journal.pone.0313432)
Supplement: Attachment — Submitted filename: Reviewer comments.pdf [file pone.0313432.s002.pdf]

Reviewer comments:

Overall, this is a clear, organized, and well-written study. I expect it to be a really valuable addition to knowledge.

Introduction is relevant and satisfactory information about the topic is presented in an organized way, yet, the researcher needs to add more previous studies mainly related to prediction and preventive measures taken by mothers worldwide, not only in the African region.

On page no 3: I suggest using "implications of study" instead of discussion. Also, the researcher uses the term (interview to collect the qualitative data). It is better to use the term (Focus group discussing: FGD) as a preferable technique for collection of this type of data.

Page 6: Researchers must add objective(s) related to (protocol) that researchers seek to achieve and develop.

The methods and materials are generally appropriate. However, more elaboration in the analysis of the qualitative data is needed, and researchers should provide justification: Why only two districts were chosen and how many districts are there in the country under study, as well as why only four health facilities?

Page no 8: more expansion why researchers chose both (full-term & preterm neonates) as sample, it is recognized that heat loss risk factors differ between premature and full-term neonates.

Data collection tools: Researchers need to add how to test the validity and reliability of data collection tools

Researchers will take (15) post-natal mothers as samples, so they need to add what precautions will be taken in case of postnatal mothers' withdrawal from the study.

Some references need to be updated

In summary: Revise and resubmit
